# Comparison of frontal QRS-T angle of patients with nasal septal deviation with healthy controls

**DOI:** 10.1186/s12872-023-03421-6

**Published:** 2023-08-23

**Authors:** Olga Bayar Kapıcı, Sabri Abuş, Selçuk Ayhan, Mehtap Koparal, Hakan Kaya

**Affiliations:** 1 Department of Radiology, Adiyaman University Education and Research Hospital, Adiyaman, Turkey; 2 Department of Cardiology, Adiyaman University Education and Research Hospital, Adiyaman, Turkey; 3 Department of Otorhinolaryngology, Adiyaman University Education and Research Hospital, Adiyaman, Turkey

**Keywords:** Nasal septal deviation, Frontal QRS-T angle, Cardiovascular disease risk

## Abstract

**Background:**

This study compares frontal QRS-T angle (fQRS-T) in electrocardiogram (ECG) examinations of people with nasal septal deviation (NSD) with healthy controls (HC).

**Methods:**

Eighty-two patients whom a radiologist with paranasal computed tomography definitively diagnosed with NSD were included in the study. 101 individuals without NSD were selected as HC.

**Results:**

Compared to the HC group, the fQRS-T in was considerably wider in patients with NSD (p < .001). According to Spearman correlation analysis, fQRS-T and NSD angle, and platelet lymphocyte ratio (PLR) were significantly correlated (p = .021, p < .001, and p = .003, respectively). In linear regression analysis where the fQRS-T was taken as a dependent variable, NSD angle and PLR predicted the fQRS-T significantly and positively (F(5.76) = 8.451, R2 = 0.357, Adjusted R2 = 0.315 and p < .001).

**Conclusion:**

In this study, fQRS-T was significantly higher in patients with NSD. In future studies, fQRS-T can be compared before and after septoplasty in NSD patients.

## Introduction

Nasal septal deviation (NSD) is a common anatomical condition where the nasal septum, which is the cartilage and bone structure that divides the left and right nasal passages, is abnormally shifted or curved to one side of the nose. This deviation can lead to various nasal symptoms and can contribute to breathing difficulties, chronic nasal congestion, recurrent sinus infections, and other related issues. NSD is highly prevalent, and in a considerable number of cases, they do not cause any noticeable symptoms. However, in some individuals, a deviated nasal septum can lead to a subjective feeling of nasal blockage and congestion. Additionally, objective assessments may reveal increased nasal resistance, reduced cross-sectional area of the nasal passages, and altered airflow patterns [1]. Chronic nasal obstruction, which can be caused by NSD, may lead to a state of chronic hypoxia (reduced oxygen levels) during sleep due to difficulties in breathing through the nose. This hypoxia may trigger various physiological responses in the body, including increased sympathetic nervous system activity, elevated blood pressure, and changes in heart rate. These changes could potentially contribute to cardiovascular strain and affect overall cardiovascular health [2]. Identifying people with NSD at high risk of cardiovascular disease may help prevent possible cardiovascular disorders in these individuals.

Cardiac problems associated with upper airway obstruction have been investigated already, and findings have suggested causality between upper airway obstruction and arrhythmias [3]. Increased prooxidant stress, increased sympathetic activity, and excessive adverse intrathoracic tension changes are linked to cardiovascular outcomes in patients who have upper airway obstruction. One of the main factors behind upper airway obstruction is NSD. P wave dispersion has been documented in individuals with adenotonsillar hypertrophy and obstructive sleep apnea [4].

People with NSD may apply to the cardiology outpatient clinic with complaints such as palpitation, shortness of breath, and feeling of suffocation. Electrocardiography (ECG) is taken on these people, and routine blood tests are examined. In addition, people with NSD may apply to the otolaryngology outpatient clinic for nasal obstruction, inability to breathe, burning in the nose, and dry mouth [5]. The fQRS-T angle (frontal QRS-T) is a simple, straightforward and distinctive ECG parameter derived from surface ECG without requiring any sophisticated software application. The mean angle difference between T wave axes and the mean QRS is known as the fQRS-T. The fQRS-T is a new parameter and provides valuable details concerning cardiac repolarization. Several investigations have identified fQRS-T may anticipate developing cardiovascular problems in various samples. It has been established that this parameter is related to arrhythmia and sudden cardiac death [6]. Currently, fQRS-T has not been extensively examined in people with NSD.

Previous studies have shown an increased risk of cardiovascular disease in NSD patients. However, fQRS-T, which predicts cardiovascular disease risk, has not been previously evaluated in NSD patients. In this study, our aim is to examine whether fQRS-T differs between NSD patients and healthy controls (HC).

## Material and method

### Study design and study group

**Fig. 1:**
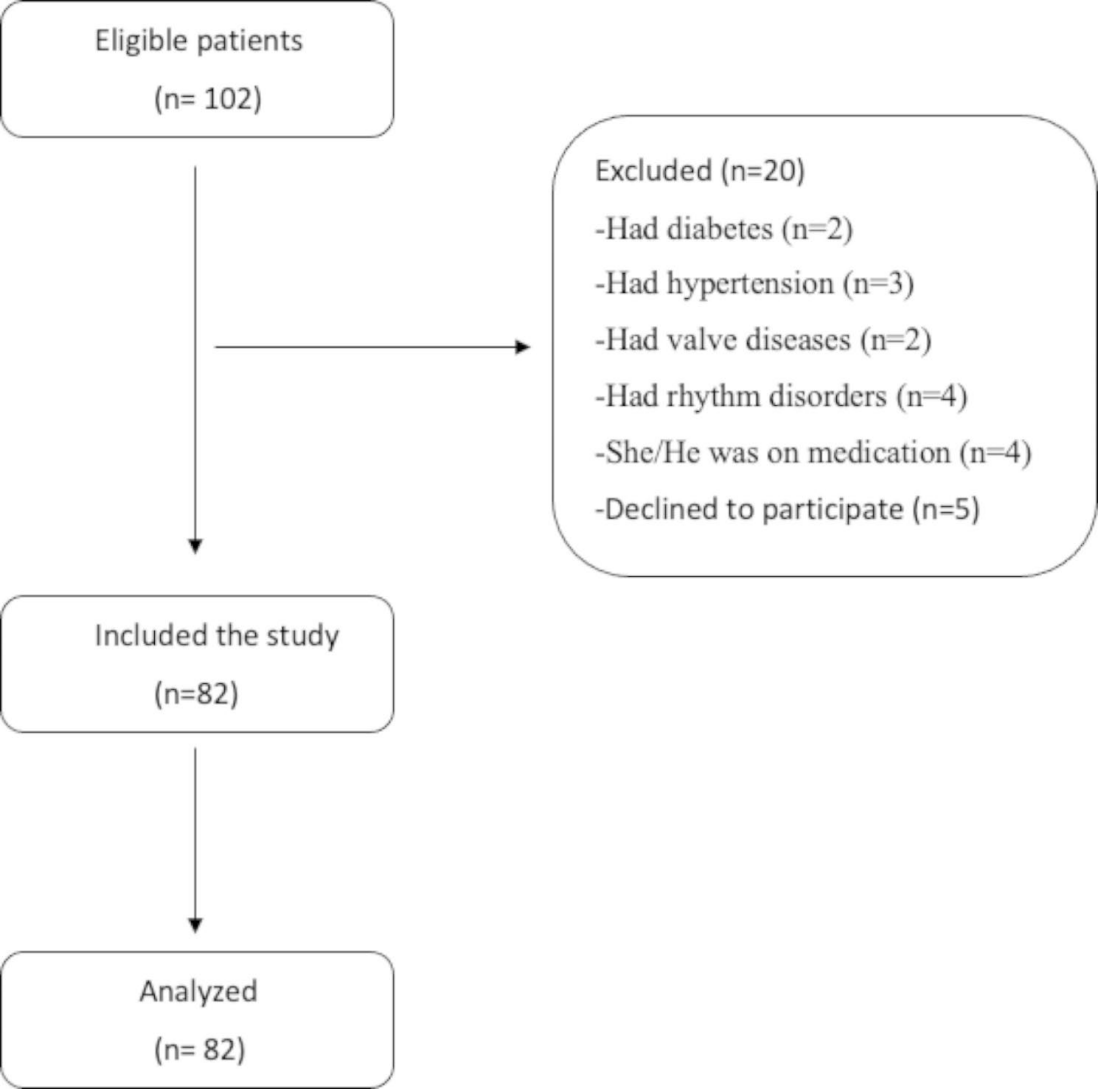
Flow-chart illustration of the study’s sample

It is a descriptive cross-sectional study. Helsinki Declaration guidelines were followed during the study. The regional ethics committee granted permission for this study (Decision number: 2022/3 − 2). The sample size was calculated as 80 using G*Power (3.1 Version, Dusseldorf, Germany) (The power of test: 0.8, alpha significance level: 0.05, Cohen’s d effect size: 0.58). The flow-chart of the study is shown in Fig. 1. Of the 102 NSD patients eligible for the study, 82 patients remained after exclusion criteria. Eighty-two patients whom a radiologist with paranasal computed tomography definitively diagnosed were included in the study. One hundred one people who shared similar characteristics to the study group including age and the gender but did not have any disease were selected to be the control group. Data on the subjects’ ages, genders, smoking habits, hemogram values, biochemistry, and ECG values were collected. By dividing the participant’s weight by the square of their height, the body mass index (BMI) was calculated.

The medical records of the patients were analyzed in the hospital file system. Paranasal sinüs computed tomography (PNS CT) and ECG were taken in patients with difficulty in breathing, sleeping, blocked nose, and speech alteration. NSD patients were identified. PNS CT and ECG were taken during the examinations of these patients in the otorhinolaryngology and cardiology outpatient clinic.

Exclusion criteria included sinusitis, allergic rhinitis, concha bullosa, and obstructive inferior nasal concha hypertrophy, as well as velopharyngeal and hypopharyngeal obstruction greater than 50% and tonsil hypertrophy ranked Grade 3 or 4 on the Brodsky scale. Those with diabetes, hypertension, heart failure, coronary artery disease, lung disease, valve diseases, rhythm disorders, abnormal cardiac conduction, and pacemakers were also excluded. NSD patients and control group did not use any medication.

### Computerized tomography examination

An imaging expert (radiologist) observed and categorized the NSD’s appearance on PNS CT pictures. CT images were retrospectively evaluated. 64-slice multislice CT scanner was used to obtain CT scans (Toshiba Medical Systems). CT examinations were performed in the face down stance with the head hyperextended. Coronal CT images with a adjacent, 1 mm-thick slice directed perpendicular to the hard palate were acquired. The bone procedure with window width (350/2700 HU) was employed to generate accurate visuals. The scanning parameters were as follows: 120 kV, 150 mAs, Ti: 1.0.

In diagnosing nasal septal deviation, computed tomography (CT) is a valuable imaging technique that provides crucial information to clinicians. The CT criteria used for this purpose include the following:

1. Deviation Angle: The degree of deviation from the midline of the nasal septum is measured in degrees. This parameter helps quantify the extent of displacement.
2. Quadrants Affected: The CT scan description includes the affected quadrants of the nose, such as anterior, posterior, superior, or inferior. This information helps localize the deviation within the nasal cavity.
3. Severity: Based on the deviation angle and its impact on nasal airflow, the severity of the deviation is classified as mild, moderate, or severe. This classification aids in determining the clinical significance of the deviation.
4. Associated Changes: The CT scan identifies any accompanying changes in the nasal cavity, which could include hypertrophy (enlargement) of the nasal turbinates or the presence of nasal polyps. These findings are crucial for a comprehensive assessment.
5. Cross-sectional Visualization: CT images provide multiple cross-sectional views of the nasal septum. This visualization allows the physician to thoroughly examine the deviation from various angles, aiding in a detailed evaluation [7].

### Nasal septal deviation classification

The NSD was assessed based on category system by Mladina and Dreher. Types 1 and 2 are described by Mladina as deviations in the cartilaginous portion of the nasal septum in the area of the nasal valve. Type 3 is shaped like a C and is situated close to the middle turbinates in the nasal cavity. Type 4 resembles the letter S with its two curving surfaces. Anterior curve is closer to the nasal valve. On the other hand, the posterior curvature is closer to the nasal cavity. type 5 contains a bony spur that can be detected inside the bony septum. The type 6 has a gutter on one side and a protuberance on the other, aligned to the horizontal plate. Type 7 is the mix of 2 or more of above mentioned types [8].

**Fig. 2:**
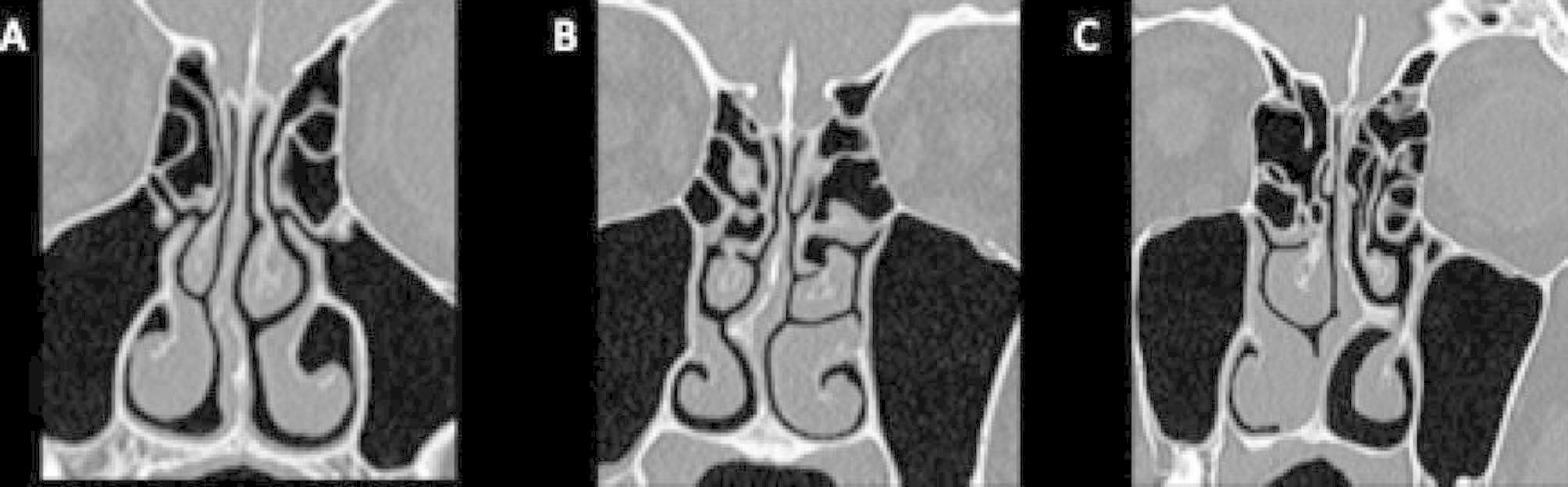
Dreher Classification of NSD on CT. **Notes**: A:Grade I, B:Grade II, C: Grade 3

According to Dreher et al., the NSD types were classified into three groups. There are three types of NSD: Type I: mild (NSD less than 50% of the distance between the midline septum and the lateral wall), Type II: moderate (NSD greater than 50% that distance but it does not touch the lateral wall), and Type III: serious (NSD touching the lateral side wall) [9]. Dreher grade system pictured in Fig. 2.

**Fig. 3:**
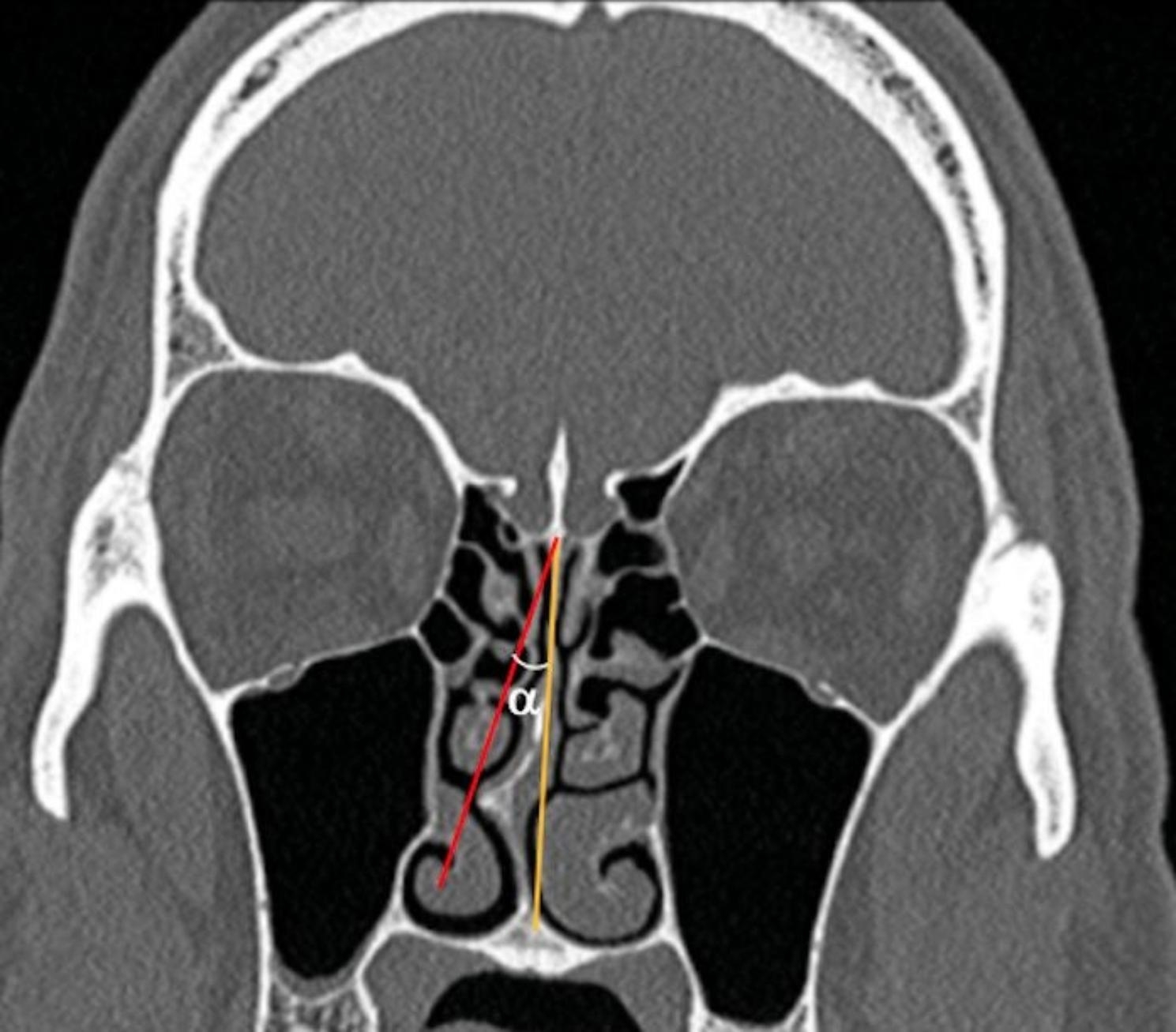
Measurement of NSD Angle on CT

We also measured the highest angle of the NSD angle. A single coronal CT section was used to measure the NSD angle. NSD angle measurement was carried out using the standard CT technique. A line of reference was used to measure the angle between two points. The midline was the first line that joined the cribriform plate criteria and originated at the septum insertion in the maxillary crest. The second line has been drawn beginning at the place where the cribriform plate and the perpendicular intersected. Figure 3 shows the calculation of the NSD angle.

### Electrocardiogram examination

12-lead ECG recordings were taken from all participants. These ECG recordings were evaluated by 2 cardiologists with at least 10 years of experience. Heart rate, QT interval, corrected QT interval, QRS interval and fQRS-T angle were acquired from ECG data. Corrected QT interval (QTc) was derived through Bazett’s equation [10]. The fQRS-T was calculated using the QRS axis and T axis difference on the ECG.

### Laboratory examination

Venous blood examinations of the participants were performed. The biochemistry laboratory examined albumin, CRP, total cholesterol, HDL cholesterol (HDL-C), LDL cholesterol (LDL-C), and fasting triglycerides. Moreover, hemogram analysis looked at hemoglobin levels as well as the number of white blood cells (WBC), neutrophils, lymphocytes, monocytes, basophils, eosinophils, and platelets. The monocyte-HDL-C ratio (MHR), number of platelet lymphocytes (PLR), CRP-albumin ratio (CAR), neutrophils-lymphocytes ratio (NLR), and monocytes-lymphocytes ratio (MLR) were all calculated. The ratio of triglycerides to HDL-C was logarithmized to determine the atherogenic index of plasma (AIP).

### Statistical analysis

SPSS version 26.0 was used to conduct the statistical analysis of the data gathered (SPSS Inc., Chicago, IL, U.S.A.). Standard deviation and mean values were used to explain numerical data, whereas percentages were used to demonstrate non-numerical data. An exploration of data pattern analysis was implemented by means of the Kolmogorov-Smirnov test. Independent sample t-tests were applied for the numerical data comparison, whereas Mann-Whitney U tests were used for the numerical data that were not continuous. To evaluate the quality of the research group’s qualitative data, chi-square analyses were conducted.Correlation analysis was accomplished through Spearman correlation. P values smaller than 0.05 are taken to be statistically significant.

## Results

**Fig. 4:**
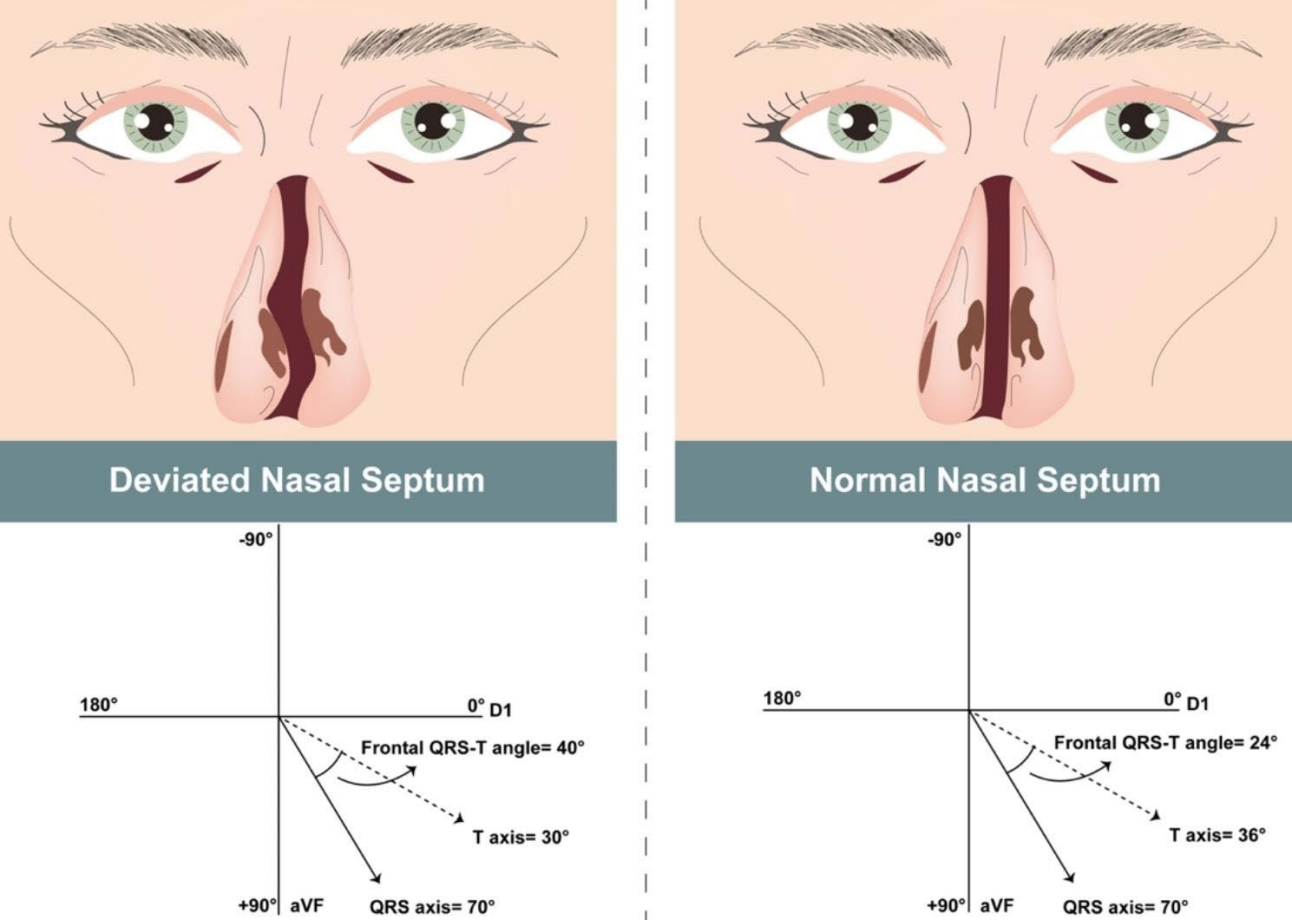
Schematic representation of fQRS values of NSD patients and HC group

82 NSD patients and 101 HC groups were enrolled in the study. Average ages and gender distribution between the NSD and HC groups did not vary substantially (p = .852, p = .60, respectively) (Table 1). (p = .852, p = .60, accordingly) (Table 1). BMIs of healthy controls and OCD patients were similar (p = .586). Patients with OCD and healthy controls did not differ significantly (p = .546) in terms of smoking status. (p = .546). The comparison of the ECG data of the NSD patients and the HC group is shown in Table 2. As a result, compared to the HC group, the fQRS-T was substantially greater in NSD patients. In Fig. 4, fQRS-T values of NSD patients and HC group are shown schematically.

**Table 1:** Sociodemographic characteristics of the patient and control groups

	NSD Group n = 82	HC Group n = 101	*p* value
Gender			
Male n (%)	52 (63.4)	50 (49.5)	.060^a^
Female n (%)	30 (36.6)	51 (49.5)	
Age (M + SD)	27.60±9.54	27.83±6.81	.852^b^
Smoking n (%)	28 (34.1)	31 (30.7)	.546^a^
BMI, kg/m^2^ (M + SD)	27.1 ± 4.89	26.8 ± 5.3	0.586^b^

NSD, Nasal septal deviation; HC, healthy controls, BMI, body mass index

a: *p* value according to chi-square analysis b: *p* value according to student t test. *p* < .05 was accepted as statistical significance value

**Table 2:** Comparison of electrocardiogram data of patient and control groups

	NSD Group n = 82 (M ± SD)	HC Group n = 101 (M ± SD)	*p* value
Heart rate, bpm	81.09 ± 16.23	78.49 ± 12.84	.767^2^
QRS, msec	89.2 ± 8.78	89.86 ± 8.14	.941^2^ .823^1^
QT, msec	362.14 ± 30.92	363.14 ± 29.30	
QTc, msec	420.07 ± 34.64	404.66 ± 26.07	.904^2^
fQRS-T	39.65 ± 25.47	24.38 ± 19.12	***< .001*** ^***2***^

NSD, Nasal septal deviation; HC, healthy controls; fQRS-T: frontal QRS-T angle

^1^Student’s t test was used. ^2^ Mann-Whitney U test was used. *p* < .05 was accepted as statistical significance value

Comparison of lab data of NSD patients and the HC group is summarized in Table 3. Accordingly, NSD patients’ albumin and hemoglobin levels were substantially greater than those of the HC group (p = .016 and p = .015, respectively). Fasting triglycerides, total cholesterol, HDL cholesterol, LDL cholesterol, and all four were significantly greater in the patients with NSD as compared to the HC group (p = .023, p < .001, p < .001, and p = .004, respectively). In comparison to the HC group, the number of basophils in patients with NSD was considerably lower (p 0.001). In comparison to the HC group, the eosinophils number, MHR, and AIP were all substantially higher in patients with NSD (p = .026, p < .001, and p < .001, respectively).

**Table 3:** Comparison of lab data of patient and control groups

	NSD Group n = 82 (M ± SD)	HC Group n = 101 (M ± SD)	*p* value
Albumin, mg/dL	4.33 ± 0.24	4.21 ± 0.62	***.016*** ^***2***^
Hemoglobin, mg/dL	14.84 ± 1.80	14.29 ± 2.02	***.015*** ^***2***^
Total-C, mg/dL	181.91 ± 37.86	170.98 ± 39.85	***.023*** ^***2***^
HDL-C, mg/dL	48.20 ± 15.22	65.90 ± 17.39	***< .001*** ^***2***^
LDL-C, mg/dL	103.11 ± 28.37	78.79 ± 30.88	***< .001*** ^***1***^
Fasting Triglyceride, mg/dL	151.03 ± 82.42	128.81 ± 99.74	***.004*** ^***2***^
WBC, 10^3^/µL	7.68 ± 1.82	8.12 ± 2.17	.169^2^
Neutrophil, 10^6^/µL	4.47 ± 1.36	4.81 ± 1.69	.150^1^
Lymphocyte, 10^3^/µL	2.40 ± 0.76	2.63 ± 1.03	.099^2^
Monocyte, 10^3^/µL	0.54 ± 0.18	0.53 ± 0.18	.611^2^
Basophil, 10^3^/µL	0.04 ± 0.03	0.10 ± 0.10	***< .001*** ^***2***^
Eosinophil, 10^3^/µL	0.20 ± 0.16	0.16 ± 0.16	***.026*** ^***2***^
Platelet, 10^3^/µL	255.77 ± 66.73	244.53 ± 53.82	.229^2^
CRP, mg/dL	0.20 ± 0.03	0.22 ± 0.10	.748^2^
NLR	2.02 ± 0.91	2.07 ± 1.08	.788^2^
MLR	0.24 ± 0.10	0.22 ± 0.12	.365^1^
PLR	119.99 ± 56.49	105.15 ± 46.69	.089^2^
CAR	0.046 ± 0.008	0.061 ± 0.066	.149^2^
MHR	0.012 ± 0.005	0.007 ± 0.004	***< .001*** ^***2***^
AIP	0.45 ± 0.30	0.07 ± 0.49	***< .001*** ^***1***^

NSD, Nasal septal deviation; HC, healthy controls; Total-C, total cholesterol; HDL-C, high-density cholesterol; LDL-C, low-density cholesterol; WBC, white blood cell; CRP, C-reactive protein; NLR; neutrophil lymphocyte ratio; MLR, monocyte lymphocyte ratio; PLR, platelet lymphocyte ratio; CAR, CRP albumin ratio; MHR, monocyte HDL-C ratio; AIP, atherogenic index of plasma

^1^Student’s t test was used. ^2^ Mann-Whitney U test was used. *p* < .05 was accepted as statistical significance value.

According to Spearman correlation analysis, fQRS-T and NSD angle, and PLR were significantly correlated (p = .021, p < .001, and p = .003, respectively) (Table 4). In linear regression analysis where the fQRS-T was taken as a dependent variable, NSD angle and PLR predicted the fQRS-T significantly and positively (F(5.76) = 8.451, R2 = 0.357, Adjusted R2 = 0.315 and p < .001) (Table 5).

**Table 4:** Spearman Correlation Analyzes of Frontal QRS-T Angle with NSD Angle, Age and Lab Parameters in Patients with NSD

	fQRS-T	NSD Angle
NSD Angle	***r = .254***	N/A
	***p = .021***	
Age	r=-.076	r=-.062
	p = .303	p = .580
MHR	r = .043	r=-.022
	p = .567	p = .847
AIP	r = .103	r=-.023
	p = .166	p = .841
PLR	***r = .215***	r = .073
	***p = .003***	p = .512

fQRS-T, frontal QRS-T angle; NSD, nasal septal deviation; MHR, monocyte HDL-C ratio; AIP, atherogenic index of plasma; PLR, platelet lymphocyte ratio

Spearman correlation test was used. *p* < .05 was accepted as statistically significant

**Table 5:** Linear Regression Analyzes of Frontal QRS-T Angle in NSD Patients

					%95 CI	
	B	Beta	t	*p*	Lower	Upper
NSD Angle	1.561	0.211	1.930	.***037***	− 0.050	3.171
Dreher Grade	4.316	0.127	1.242	0.218	-2.603	11.236
Mladina Class	1.500	0.152	1.462	0.148	− 0.544	3.544
PLR	0.124	0.274	2.933	***0.004***	0.040	0.207
Age	1.722	0.96	2.322	0.198	-1.668	4.264
Constant	-21.870		-2.118	0.037		

NSD, Nasal septal deviation; PLR, platelet lymphocyte ratio; CI, confidence interval

Lineer regression analyses was used. *p* < .05 was accepted as statically significance. F_(5,76)_ = 8.451, R^2^ = 0.357, Adjusted R^2^ = 0.315 and p < .001

It was not significant to distinguish the groups regarding fQRS-T according to the Kruskal-Wallis test for Mladina calcification (Table 6). According to the Dreher classification, the fQRS-T differed significantly between the groups (p = .026) (Table 7). When compared to Dreher grade 1 and grade 2 NSD patients, Dreher grade 3 NSD patients had a substantially greater fQRS-T according to the Mann-Whitney U test analysis. (p = .013 and p = .017). There was no significant difference in fQRS-T between Dreher grade 1 and grade 2 NSD patients (p = .427).

**Table 6:** Frontal QRS-T Angle in NSD Types According to Mladina Classification

	n (%)	fQRS-T (M ± SD)
Type 1	35 (42.6%)	31.80 ± 14.56
Type 2	2 (2.4%)	50.50 ± 3.53
Type 3	2 (2.4%)	46.50 ± 41.71
Type 4	7 (8.5%)	42.57 ± 26.01
Type 5	11 (13.4%)	37.63 ± 26.87
Type 6	2 (2.4%)	13.00 ± 12.72
Type 7	23 (28%)	52.47 ± 33.27
Kruskall-Wallis		*p* = .145

fQRS-T, frontal QRS-T angle

Kruskall-Wallis test was used. *p* < .05 was accepted as statically significance

**Table 7:** Frontal QRS-T Angle in NSD Types According to Dreher Classification

	n (%)	fQRS-T (M ± SD)
Type 1	45 (54.8%)	33.75 ± 23.71
Type 2	24 (30%)	36.84 ± 20.75
Type 3	13 (15.8%)	60.30 ± 34.07
Kruskall-Wallis		***p = .026***

fQRS-T, frontal QRS-T angle

Kruskall-Wallis test was used. *p* < .05 was accepted as statically significance

## Discussion

The primary outcome of this research is that the fQRS-T is statistically wider in patients with NSD. Moreover, fQRS-T correlated with NSD angle.

NSD is one of the most frequent etiologies of incomplete or total obstruction of chronic upper airway obstruction. Nasal airflow resistance accounts for about half of respiratory airflow resistance; therefore, slight variations in the nasal opening can change overall airway resistance. Deviation of the nasal septum may cause chronic mucosal irritation and postnasal drip, as well as increased airway resistance [11]. The relationship of upper airway obstruction with cardiovascular events such as pulmonary hypertension, cardiac arrhythmias, and mortalities associated with cardiovascular disease has been studied previously [12]. Furthermore, NSD is a causal element for sleep apnea syndrome [11]. While the relationship between sleep apnea syndrome and ventricular arrhythmias has been widely examined, studies investigating the relationship between NSD and ventricular arrhythmias are few [13–16]. This is the first study in the literature to evaluate fQRS-T in NSD.

A 24-hour rhythm holter was used by Derin et al. to examine arrhythmia in patients with NSD before and after a nasal septoplasty operation and they observed that ventricular and supraventricular extrasystoles were decreased in postoperative patients. They suggested that this was attributed to the relief of upper airway obstruction and the reduction of sustained hypoxemia and autonomic nerve dysfunction due to chronic airway irritation [17]. Taşolar et al. found a marked shortening in QT dispersion and QTc dispersion interval after septoplasty and interpreted that septoplasty operation would lower the possibility of cardiac arrhythmia [4]. Kılıçarslan et al. looked into how the Tp-e/QT ratio and obstructive sleep apnea syndrome patients’ Tp-e intervals related to one another. They observed that these measurements were extended and positively correlated with the degree of sleep apnea syndrome [18].

In addition to indicating poor cardiac conduction, the QRS-T angle is a characteristic ECG sign for the ventricular repolarization index. The spatial QRS-T angle is an angle formed by the vectors of the QRS and T waves in 3-D space, and also the fQRS-T angle is the projection of this angle on the frontal plane. The spatial and frontal QRS-T angles are associated with cardiovascular events and mortality, as stated by a number of observational studies [19, 20]. One study in a significant population-based cohort has come to the conclusion that a high spatial QRS-T angle (> 130°) is a predictor of impending lethal cardiovascular events, specifically sudden cardiac death. The spatial-QRS-T angle, interestingly, was the leading indicator when compared to both conventional cardiovascular disease indicators and common ECG risk markers including left ventricular hypertrophy, left bundle branch block, T wave inversion, and QTc interval [21]. Participants with an atypical spatial QRS-T angle (136° for men and 121° for women) and without the clinically evident cardiovascular disease had a twice as high risk of cardiovascular death, including suspected sudden death [22].

fQRS-T is associated with cardiovascular conditions. A large survey of the general Finnish community found that those with a high fQRS-T (> 100°) had a three-fold higher risk of sudden death within 30 years [19]. In another cohort study, the incidence of cardiovascular diseases was higher in adults without cardiovascular disease but with a high fQRS-T [20]. In addition to general population studies, studies investigating the causal link between fQRS-T and cardiovascular events in individuals with cardiovascular disease have also been conducted. For example, in patients with implantable defibrillators, the initially measured fQRS-T is a significant marker of appropriate ICD therapy, potentially as an indicator of malignant ventricular arrhythmias [23, 24].

Obstructive respiratory events cause intermittent hypoxemia, increased ventricular load, and impaired sympathovagal balance [25]. This may aggravate nocturnal ventricular arrhythmogenicity, presumably via ion channel dysfunction [26]. Irregular breathing during sleep has been demonstrated in these patients as dynamic changes in ECG patterns of ventricular repolarization during sleep and attenuation in continuous positive airway pressure [18]. These events may contribute to sustained long-term instability, particularly in cardiac structural remodeling and myocardial action potential, which increases the chances of sudden death.

In the study, triglyceride, total cholesterol, LDL cholesterol were higher and HDL cholesterol was lower in NDS patients. Although there is no data in the literature that NSD can affect triglyceride and cholesterol levels, changes in lipid and cholesterol metabolism may occur if NSD causes sleep-respiratory disorders such as obstructive sleep apnea.

### Limitations

Conducting this study in a larger patient population and evaluating other data showing all markers of ventricular repolarization on the ECG, such as Tp-e, Tp-e\QT, could provide a better assessment. One of the limitations of our study is the absence of electrolyte levels such as calcium and magnesium and thyroid function tests, which potentially alter the QT interval and ventricular repolarization. Since the normal ranges of fQRS are unknown, prospective longitudinal axis studies are needed to predict the risk of cardiovascular disease in NSD patients. The major limitation of this study is that fQRS-T comparison before and after septoplasty was not made. Possible fQRS-T variation before and after septoplasty may be helpful in understanding causality. Not using rhinomanometry to evaluate nasal obstruction is a limitation. In many cases, the presence of NSD alone does not necessarily correlate with subjective discomfort, difficulty in breathing, sleeping, or speech alteration. Therefore, using the Nasal Obstruction Symptom Evaluation (NOSE) scale would have allowed us to make more objective interpretations in the evaluation of nasal obstruction.

## Conclusion

In this study, fQRS-T was significantly higher in patients with NSD. In this study, NSD angle and fQRS-T were correlated, and according to Dreher grading a significant difference was revealed in fQRS-T between the groups. The higher fQRS-T in those with Dreher Type 3 NSD may indicate the relationship between degree of NSD and increased fQRS-T. It was found that the fQRS-T increased as the degree of NSD increased. In future studies, fQRS-T can be compared before and after septoplasty in NSD patients.

## Data Availability

The datasets used and analyzed during the current study are available from the corresponding author on reasonable request.
